# Comparative evaluation of the modulatory role of 1,25-dihydroxy-vitamin D_3_ on endoplasmic reticulum stress-induced effects in 2D and 3D cultures of the intestinal porcine epithelial cell line IPEC-J2

**DOI:** 10.1186/s40104-024-01112-6

**Published:** 2024-11-10

**Authors:** Gaiping Wen, Klaus Eder, Robert Ringseis

**Affiliations:** 1 Institute of Animal Nutrition and Nutrition Physiology, Justus Liebig University Giessen, Heinrich-Buff-Ring 26-32, Giessen, 35392 Germany; 2 Center for Sustainable Food Systems, Justus Liebig University Giessen, Senkenbergstraße 3, Giessen, 35390 Germany

**Keywords:** 1,25-Dihydroxy-vitamin D_3_, 3D cell culture, Endoplasmic reticulum stress, Intestinal barrier, Pig

## Abstract

**Background:**

The use of conventional two-dimensional (2D) culture of the porcine intestinal epithelial cell (IEC) line IPEC-J2 in animal nutrition research has the disadvantage that IEC function is studied under unphysiological conditions, which limits the ability of transferring knowledge to the in vivo-situation. Thus, the aim of the present study was to establish a more convincing and meaningful three-dimensional (3D) culture of IPEC-J2 cells, which allows to study cell function in a more tissue-like environment, and to compare the effect of the endoplasmic reticulum (ER) stress inducer tunicamycin (TM) on ER stress indicators and the expression of tight junction proteins (TJP), inflammatory and apoptosis-related genes and the modulatory role of 1,25-dihydroxy-vitamin D_3_ (1,25D_3_) on these parameters in 2D and 3D cultures of IPEC-J2 cells.

**Results:**

A published protocol for 3D culture of Caco-2 cells was successfully adopted to IPEC-J2 cells as evident from fully differentiated 3D IPEC-J2 spheroids showing the characteristic spherical architecture with a single layer of IPEC-J2 cells surrounding a central lumen. Treatment of 2D IPEC-J2 cells and 3D IPEC-J2 spheroids with TM for 24 h markedly increased mRNA and/or protein levels of the ER stress target genes, heat shock protein family A (Hsp70) member 5 (*HSPA5*) and DNA damage inducible transcript 3 (*DDIT3*), whereas co-treatment with TM and 1,25D_3_ did not mitigate TM-induced ER stress in IPEC-J2 cells in the 2D and the 3D cell culture. In contrast, TM-induced expression of pro-inflammatory [interleukin-6 (*IL6*), *IL8*] and pro-apoptotic genes [BCL2 associated X, apoptosis regulator (*BAX*), caspase 3 (*CASP3*), *CASP8*] and genes encoding TJP [*TJP1*, claudin 1 (*CLDN1*), *CLDN3*, occludin (*OCLN*), cadherin 1 (*CDH1*), junctional adhesion molecule 1 (*JAM1*)] was reduced by co-treatment with TM and 1,25D_3_ in 3D IPEC-J2 spheroids but not in the 2D cell culture.

**Conclusions:**

The effect of 1,25D_3_ in the IPEC-J2 cell culture is dependent on the culture model applied. While 1,25D_3_ does not inhibit TM-induced expression of genes involved in inflammation, apoptosis and TJP in conventional 2D cultures of IPEC-J2 cells, TM-induced expression of these genes is abrogated by 1,25D_3_ in the more meaningful 3D IPEC-J2 cell culture model.

**Supplementary Information:**

The online version contains supplementary material available at 10.1186/s40104-024-01112-6.

## Background

The inner surface of the intestine consists of a single layer of intestinal epithelial cells (IEC) which are not only important for nutrient absorption but also provide protection of the organism against harmful molecules and bacteria by forming a physical and functional cellular barrier [1, 2]. Key structural elements of this cellular barrier are different tight junction proteins (TJP), which connect adjacent IEC and play a key role in controlling paracellular transport across the gut barrier but are also important in regulating IEC proliferation and differentiation [3]. Intestinal barrier defects resulting from intestinal inflammation are known to be associated with an altered expression and relocalization of intestinal TJP, thereby, leading to hyperpermeability (“leaky gut”) and increased translocation of microbial-associated molecular patterns (MAMP), such as bacterial endotoxins, bacterial DNA and even intact microbes [4]. Excessive levels of MAMP can overcharge the protective mechanisms of the liver, which leads to hepatic and systemic inflammation and profound metabolic derangements in almost all tissues of the organism. Thus, maintaining intestinal barrier function is essential in the prevention of metabolic health.

IEC are equipped with several stress-responsive signaling pathways including the endoplasmic reticulum (ER) stress-induced unfolded protein response (UPR) pathway. ER stress describes a state characterized by the disruption of ER functions, such as protein synthesis, folding, trafficking and degradation, and calcium ion homeostasis [5]. As a consequence of ER stress, the UPR is initiated which comprises three main transmembrane signaling pathways: PKR-like ER kinase, inositol-requiring enzyme 1α, and activating transcription factor 6. The UPR serves to restore protein homeostasis and decrease accumulation of incompletely or incorrectly folded proteins by decreasing new protein synthesis leading to a reduction of newly synthesized proteins entering the ER, but induces programmed cell death (apoptosis) in the case of irreversible ER disruption [6, 7].

Considerable evidence has been gained that vitamin D_3_ hormone, 1,25-dihydroxy-vitamin D_3_ (1,25D_3_), attenuates ER stress in many cell types. One of the decisive mechanisms underlying this effect is the anti-inflammatory action of 1,25D_3_, because inflammatory mediators are well-known stimulators of ER stress [6, 7]. Our own group has shown that 1,25D_3_ decreases the ER stress-induced inflammatory response in mammary epithelial cells [8], but 1,25D_3_ was also found to inhibit ER stress in endothelial cells [9], monocytes [10], and macrophages [11]. An inhibition of ER stress has been also reported for cultivated intestinal cells in response to different bioactive compounds [12], whereas the effect of 1,25D_3_ on ER stress and ER stress-mediated effects in intestinal cells is currently unknown. The conventional two-dimensional (2D) culture of an intestinal porcine epithelial cell line of the jejunum (IPEC-J2) is an established in vitro-model of the porcine small intestine, which is widely used in animal nutrition research to investigate the mode of action of nutrients and other bioactive substances in IEC. However, the disadvantage of the conventional 2D culture of adherent cells, such as IPEC-J2 cells, is that IEC function is studied under unphysiological conditions with limited contact between adjacent cells and the lack of interaction between cells and the extracellular matrix (ECM), which limits the ability of transferring knowledge to the in vivo-situation. Cultivation of cells in an artificial ECM, referred to as three-dimensional (3D) culture, allows to study cell function in a more tissue-like environment with more pronounced cell–cell and cell–ECM interactions than in the 2D culture [13, 14]. While the above-cited research shows an effect of 1,25D_3_ on biological processes associated with ER stress in 2D culture conditions [8–12], it is currently unknown if those effects can be also observed in 3D models of the intestinal epithelium. To address this question is particular important when studying the effect of nutrients in IEC models, such as the IPEC-J2 cell line, because 2D cultures of IEC are incomplete models of the intestinal epithelium which grow in monolayers and do not form the typical crypt-villus-structures of the small intestine. Thus, the successful establishment of a yet missing 3D culture of IPEC-J2 cells would enable researchers in the field of animal nutrition to investigate the effects of nutrients in a more appropriate in vitro-model better mimicking the in vivo-architecture of the intestinal epithelium. Thus, the aim of the present study was (1) to establish a 3D culture of IPEC-J2 cells, and (2) to compare the effect of the ER stress inducer tunicamycin (TM) on ER stress indicators and the expression of TJP, inflammatory and apoptosis-related genes and the modulatory role of 1,25D_3_ on these parameters in 2D and 3D cultures of IPEC-J2 cells.

## Methods

### 2D culture of IPEC-J2 cells

The IPEC-J2 cell line (DSMZ No.: ACC 701) was purchased from the DSMZ (Braunschweig, Germany). It represents a non-transformed, non-tumorigenic jejunal porcine epithelial cell line which can be continuously maintained in culture. The cells were cultured in Dulbecco’s Modified Eagle Medium (DMEM) growth medium supplemented with 10% heat-inactivated fetal bovine serum (FBS; both from Gibco/Life Technologies, Darmstadt, Germany), 100 U/mL penicillin, and 100 µg/mL streptomycin (Th. Geyer, Höxter, Germany) at 37 °C in a humidified atmosphere of 90% air and 10% CO_2_. Growth medium was changed every 2 d. After reaching a confluence of 70%–80%, the cells were either sub-cultivated or used for experiments. To detach and passage cells, 0.25% trypsin–EDTA (Th. Geyer, Germany) was used.

### 3D culture of IPEC-J2 cells

A thin layer of Matrigel (130 µL/well for 24-well plates and 100 µL/well for 8-well chambers; Th. Geyer) was coated on the surface of the pre-chilled culture plates and incubated at 37 °C for 30 min to allow gelation of the Matrigel. Prior to plating of the IPEC-J2 cells on the Matrigel-coated surface, an IPEC-J2 cell suspension was prepared in antibiotic-free growth medium from 2D IPEC-J2 cell culture using trypsin. Following plating, the cells were incubated at 37 °C for 20–30 min to allow the cells to attach to the Matrigel. Afterwards, a Matrigel-growth medium mixture (10% Matrigel, 90% growth medium without antibiotics, v/v) was added to the culture plates and spheroids were allowed to form over a period of 5 d during which the Matrigel-growth medium mixture was replaced every 2 d [15, 16]. The Matrigel was required in the growth medium to maintain a proper culture environment promoting efficient cell growth and differentiation. Normally, there were about 70%–80% spheroids showing the typical morphological traits of intestinal epithelium.

### Identification of morphological characteristics of IPEC-J2 spheroids

IPEC-J2 cells were seeded in 24-well plates at a density of 3 × 10^4^ cells/well and incubated on Matrigel for 5 d. The spheroids were washed 2 times with ice-cold PBS and fixed with ice-cold 4% paraformaldehyde at room temperature (RT) for 10 min. Fixation did not affect Matrigel consistence. Subsequently, the spheroids were incubated with phalloidin-TRITC (1 µg/mL in PBS) at 4 °C overnight. After washing and staining the nuclei with Hoechst (5 µg/mL in PBS) at RT for 5 min, slides were mounted in Aqua-Poly/Mount (Polysciences, Eppelheim, Germany) and analyzed using an EVOS M5000 fluorescence microscope (Thermo Fisher Scientific, Waltham, MA, USA).

### MTT cell viability assay

The 3-(4,5-dimethylthiazol-2-yl)-2,5-diphenyltetrazolium bromide (MTT) assay was used to assess cell viability in response to the two treatment compounds, TM and 1,25D_3_ (all from Sigma-Aldrich, Steinheim, Germany), as described recently [17]. IPEC-J2 cells were seeded in 96-well culture plates at a density of 1 × 10^4^ cells/well and incubated in the 2D cell culture medium. After reaching a confluence of 70%–80%, cells were treated either without (0.1% DMSO alone) or with TM or 1,25D_3_ (both dissolved in DMSO) for 24 h as indicated in the figure legends. Cell viabilities of cells treated with different concentrations of TM or 1,25D_3_ are presented relative to that of cells treated with the same vehicle (DMSO) concentration (0.1%).

### Live/Dead assay

To assess IPEC-J2 viability in response to TM and 1,25D_3_ in the 3D culture, cells were seeded in 24-well plates at a density of 2.5–3.0 × 10^4^ cells/well and incubated on Matrigel for 5 d until spheroids were formed as described above. Afterwards, the spheroids were treated either without (0.1% DMSO alone) or with TM or 1,25D_3_ for 24 h as indicated in the figure legends. After treatment, the Cyto3D Live/Dead assay kit (TheWell Bioscience, North Brunswick, New Jersey, USA) was used to determine live and dead cells according to the manufacturer's protocol.

### RNA isolation and qPCR

IPEC-J2 cells were seeded in 24-well plates at a density of 3 × 10^4^ cells/well and incubated in 2D cell culture medium until 70%–80% confluent. Subsequently, the cells were treated either without (0.1% DMSO alone) or with TM or with TM and 1,25D_3_ for 24 h as indicated in the figure legends. Isolation of total RNA, synthesis of cDNA and qPCR were performed as described recently [18]. For 3D culture, IPEC-J2 cells were seeded in 24-well plates at a density of 2.5–3 × 10^4^ cells/well and incubated on Matrigel for 5 d until spheroids were formed as described above. Following treatment of spheroids as indicated in the figure legends, spheroids were washed 2 times with ice-cold PBS followed by addition of ice-cold PBS-EDTA (5 mmol/L EDTA, 1 mmol/L sodium vanadium oxide, 1.5 mmol/L sodium fluoride in PBS). Matrigel was detached from the bottom of the 3D culture surface by gently scraping the bottom with a pipette tip. After shaking the plate for 30 min, the solution was transferred to a 1.5-mL tube and gently shaken on ice for additional 30 min. Afterwards, the spheroids were centrifuged (1,000 r/min) at 4 °C for 5 min. The spheroids were lysed with Trizol RNA extraction buffer and total RNA isolation, cDNA synthesis and qPCR were carried out as described above. Characteristics of gene-specific primers are listed in Additional file 1: Table S1. Relative mRNA levels of target genes were normalized by the mRNA level of ribosomal protein S9 (*RPS9*). Normalized mRNA levels of cells treated with DMSO alone were set to 1 and means and SD of cells of the other treatments were scaled proportionately.

### Immunoblotting

For immunoblotting experiments, IPEC-J2 cells were seeded in 6-well culture plates at a density of 1 × 10^5^ cells/well and incubated until 70%–80% confluent. Subsequently, the cells were treated either without (0.1% DMSO alone) or with TM or with TM and 1,25D_3_ for 24 h as indicated in the figure legends. The 10 μg total protein was separated on 10% SDS-PAGE and electro-transferred to a nitrocellulose membrane (Pall Corp, Pensacola, FL, USA). After blocking membranes at 4 °C overnight, membranes were incubated with primary antibodies rabbit anti-HSPA5 (dilution 1:5,000; Thermo Fisher Scientific), mouse anti-DDIT3 (dilution 1:1,000; Thermo Fisher Scientific), and mouse anti-VDR (dilution 1:300; St. Cruz, Heidelberg, Germany). The primary antibody rabbit anti-GAPDH (1:2,500; Abcam, Cambridge, UK) was incubated as a reference protein to control for adequate normalization at RT for 2 h. The membranes were washed, and then incubated with horseradish peroxidase-conjugated secondary antibodies anti-rabbit-IgG (dilution 1:10,000; Sigma-Aldrich) or anti-mouse-IgG (dilution 1:10,000; St. Cruz) at RT for 2 h. Afterward, blots were developed using ECL Plus (GE Healthcare, München, Germany). The signal intensities of specific bands were detected with a Bio-Imaging system (Syngene, Cambridge, UK) and quantified using Syngene GeneTools software (nonlinear dynamics; Syngene). Normalized protein levels of cells treated with DMSO alone were set to 1 and means and SD of cells of the other treatments were scaled proportionately.

### Immunocytochemistry (ICC) of 2D culture of IPEC-J2 cells

IPEC-J2 cells were seeded in 24-well plates at a density of 3 × 10^4^ cells/well. After reaching a confluence of 70%–80%, the cells were treated either without (0.1% DMSO alone) or with TM or with TM and 1,25D_3_ for 24 h as indicated in the figure legends. Subsequently, cells were rinsed 2 times with ice-cold PBS and fixed with 4% paraformaldehyde at RT for 10 min. To stop fixation 10 mmol/L glycine (in PBS) was added and incubated at RT for 10 min. Afterwards, cells were washed 2 times with PBS at RT and incubated with primary antibodies rabbit anti-OCLN (dilution 1:100), rabbit anti-ZO (dilution 1:1,000), mouse anti-E-cadherin (dilution 1:100) (all from Thermo Fisher Scientific), and rabbit anti-α-Tubulin (dilution 1:50; Cell Signaling Technology, Danvers, MA, USA) or mouse anti-α-Tubulin (dilution 1:50; Thermo Fisher Scientific) as a reference protein in 0.25%–0.5% Triton X-100, 10% normal goat serum in PBS at 4 °C overnight. Afterwards, cells were washed 3 times with PBS buffer at RT for 10 min and incubated with secondary antibody anti-mouse IgG Alexa Fluor 488 (dilution 1:500) and anti-rabbit IgG Alexa Fluor 594 (dilution 1:400) (both from Thermo Fisher Scientific) in 0.25%–0.5% Triton X-100, 10% normal goat serum in PBS at RT for 2 h. After 3 washing steps with PBS, nuclei were stained with Hoechst (dilution 1:10,000 in PBS; Abnova) at RT for 5 min. Finally, slides were washed 3 times with PBS at RT for 10 min and mounted in Aqua-Poly/Mount (Polysciences, Eppelheim, Germany). Stained cells were analyzed using an EVOS M5000 fluorescence microscope. At least five microscopic fields were randomly selected for the image capture at 40× magnification.

### Immunocytochemistry (ICC) of 3D culture of IPEC-J2 cells

IPEC-J2 cells were seeded in 8-well chambers at a density of 5 × 10^3^ cells/well and incubated on Matrigel for 5 d (until spheroids were formed) replacing Matrigel-medium mixture every 2 d. Subsequently, the spheroids were treated either without (0.1% DMSO alone) or with TM or with TM and 1,25D_3_ for 24 h as indicated in the figure legends, and then, rinsed 2 times with ice-cold PBS and fixed with ice-cold 4% paraformaldehyde at RT for 10 min. To stop fixation, 10 mmol/L glycine was added and incubated for 10 min. After two washing steps with 10 mmol/L glycine at RT for 10 min, IF blocking buffer [1% goat anti-mouse IgG Fab from Biozol (Munich, Germany) and 10% goat serum in IF buffer containing 0.2% Triton X-100, 0.1% BSA and 0.05% Tween 20 in PBS] was added and the slides were incubated for 1.5–2 h at RT in a humid chamber. After blocking, the spheroids were incubated with primary antibodies rabbit anti-HSPA5 (dilution 1:300), mouse anti-DDIT3 (dilution 1:50), rabbit anti-OCLN (dilution 1:100), rabbit anti-ZO (dilution 1:1,000; Thermo Fisher Scientific), mouse anti-VDR (dilution 1:50), and rabbit anti-α-Tubulin (dilution 1:50) or mouse anti-α-Tubulin (dilution 1:50) as a reference protein at 4 °C overnight. Afterwards, spheroids were washed 3 times with IF buffer at RT for 20 min, and then incubated with secondary antibody anti-mouse IgG Alexa Fluor 488 (dilution 1:500) and anti-rabbit IgG Alexa Fluor 594 (dilution 1:400) in IF buffer for 2 h at RT in a humid chamber. Subsequently, slides were washed once with IF buffer for 20 min and 2 times with PBS for 10 min at RT. Finally, nuclei were stained with Hoechst (dilution 1:10,000) for 5 min at RT. After washing, the slides were mounted in Aqua-Poly/Mount and the stained spheroids were analyzed using a fluorescence microscope as described above.

### Secreted alkaline phosphatase (SEAP) assay

IPEC-J2 cells were seeded in 96-well plates at a density of 1 × 10^4^ cells/well and incubated in 2D cell culture medium. After reaching a confluence of 60%–70%, cells were transfected with either positive control plasmid pSEAP2-control (containing a truncated placental alkaline phosphatase gene and the constitutive SV40 early promoter and enhancer) or negative control plasmid pSEAP2-Basic (containing the SEAP gene but lacking eukaryotic promoter and enhancer sequences to drive expression) using FuGENE6 (Promega, Madison, WI, USA) for 12 h. Afterwards, cells were treated either without (0.1% DMSO alone) or with TM or with TM and 1,25D_3_ for 24 h as indicated in the figure legends. After treatment, Great EscAPe SEAP Chemiluminescence Detection Kit (Takara, Germany) was used to determine the activity of secreted alkaline phosphatase (SEAP) according to the manufacturer's protocol. SEAP activity was measured with an Infinite 200M microplate reader (Tecan, Mainz, Germany).

### Statistical analysis

Statistical analysis was performed using the Minitab statistical software (Rel. 13.0, State College, PA, USA). Data from qPCR are means and SD calculated from three replicates for the same treatment of three independent experiments. Data from immunoblotting are means and SD calculated from one replicate for the same treatment of three independent experiments. Data from MTT assay are means and SD from one independent experiment performed in octuplicate. MTT assay was performed twice and both independent experiments showed similar results. Data from qPCR, immunoblotting and MTT assay was analysed by 1-factorial ANOVA. For statistically significant *F* values, individual means of the treatment groups were compared by Fisher’s multiple range test. Effects were considered significant if *P* < 0.05.

## Results

### Morphological features of IPEC-J2 spheroids

**Fig. 1:**
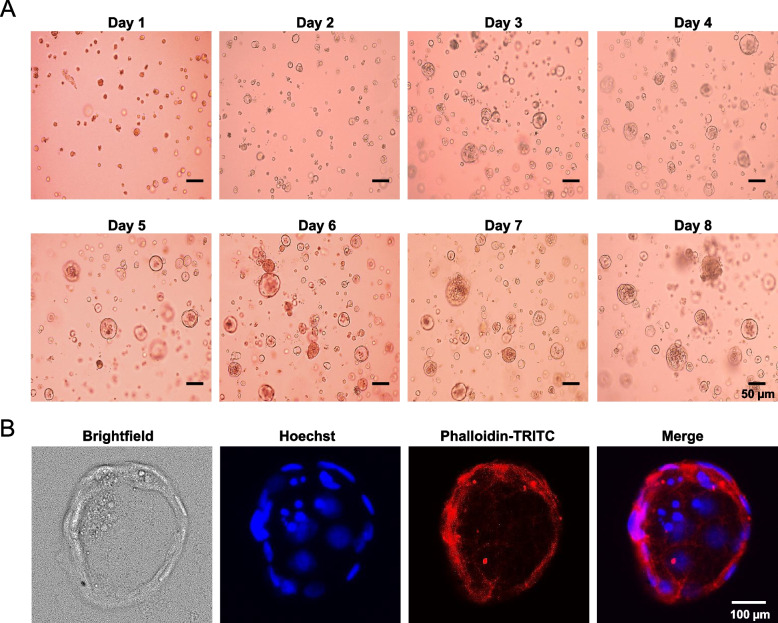
Development of IPEC-J2 spheroids during cultivation on 3D Matrigel. **A** IPEC-J2 cells were incubated on Matrigel in 3D cell culture medium for 8 d, and one representative brightfield image (10× magnification) was taken each d to show development of the spheroids. **B** Representative images from ICC staining (40× magnification) of a single differentiated IPEC-J2 spheroid after 5 d of cultivation on Matrigel in 3D cell culture medium for Hoechst detecting nuclei (blue) and Phalloidin-TRITC detecting actin filaments (red) are shown. A merged image from all three stainings and a brightfield image is also shown. The size of the spheroids is indicated by the scale bar

As shown in Fig. 1A, IPEC-J2 cells readily formed hollow spheroids starting from d 2 of cultivation in Matrigel. ICC-staining of a single IPEC-J2 spheroid from d 5 of cultivation in Matrigel demonstrates that cells had arranged as single layers of cells, visualized by Hoechst-stained nuclei, surrounding a central lumen (Fig. 1B). Proteins that are typically found at the luminal side of the cytoplasm, such as filamentous actin, visualized by Phalloidin-TRITC, predominantly localized to the cell surfaces lining the lumens of the IPEC-J2 spheroids indicating polarization of the IPEC-J2 cells (Fig. 1B). The merge image confirms the spatial arrangement of the cells in the spheroids having a central lumen (Fig. 1B). Typically, from 5 d onwards the 3D IPEC-J2 cell culture consisted of more than 70% spheroids with a single lumen, which were used for incubation experiments. In order to investigate if other cell types than enterocytes are present in the differentiated IPEC-J2 spheroids, the expression of intestinal stem cell marker G-protein-coupled receptor 5 (LGR5), goblet cell marker mucin 2 (MUC2), and enteroendocrine cell marker chromogranin A (CHGA) was measured using qPCR and ICC, respectively. However, expression of LGR5, MUC2 and CHGA was not detectable at all (data not shown).

### Effect of 1,25D_3_ and TM on the viability of IPEC-J2 cells in the 2D and the 3D cell culture

**Fig. 2:**
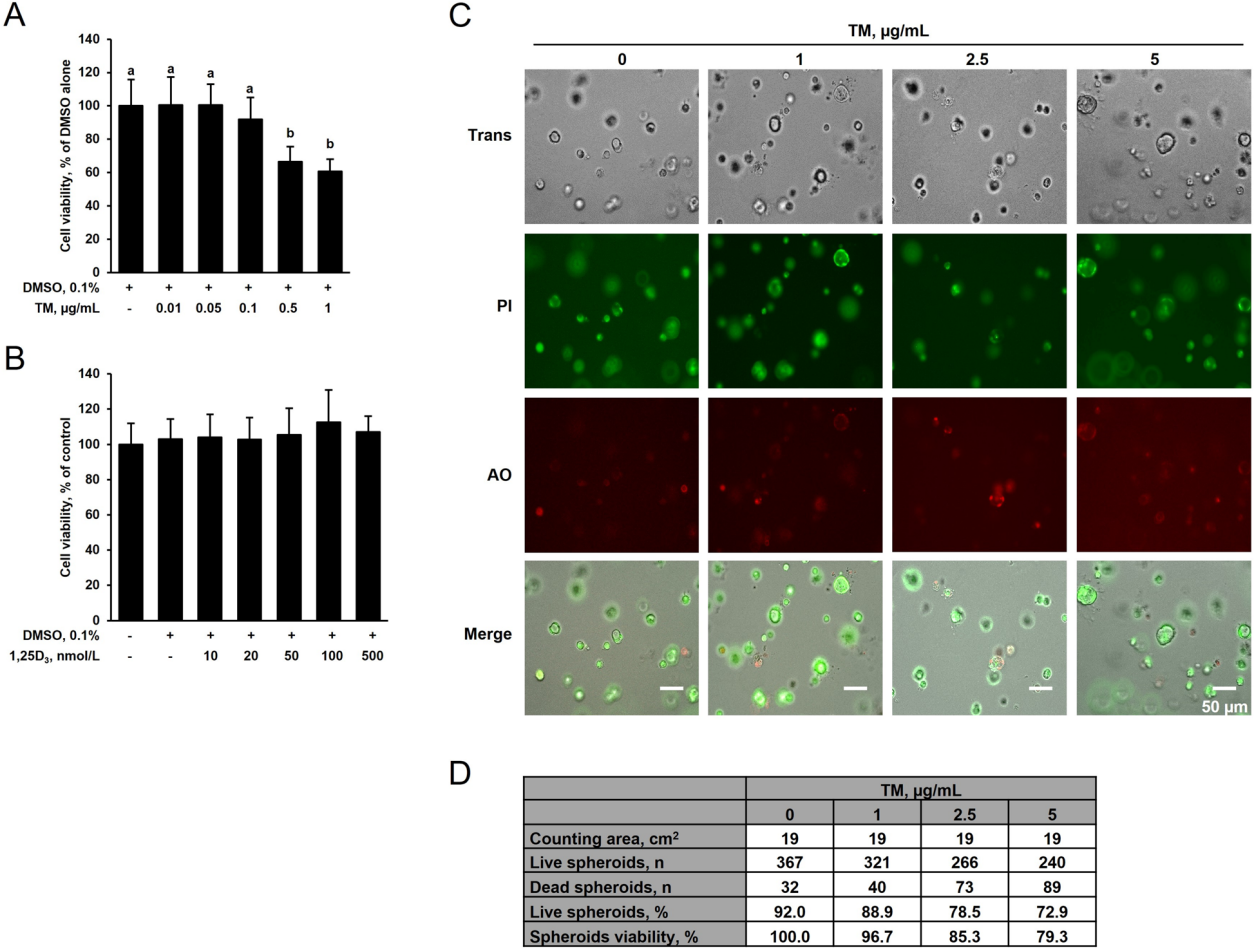
Effect of tunicamycin (TM) and 1,25-dihydroxy-vitamin D_3_ (1,25D_3_) on viability of 2D IPEC-J2 cells and 3D IPEC-J2 spheroids. For 2D culture, 70%–80% confluent IPEC-J2 cells were treated either (**A**) without (0.1% DMSO alone) or with different concentrations of TM (0.01, 0.05, 0.1, 0.5 and 1 µg/mL) or (**B**) without (0.1% DMSO alone) or with different concentrations of 1,25D_3_ (10, 20, 50, 100 and 500 nmol/L) for 24 h, and cell viability was evaluated by MTT test. **C** For 3D culture, differentiated IPEC-J2 spheroids were treated either without (0.1% DMSO alone) or with TM (1 µg/mL) or with different concentrations of TM (1, 2.5 and 5 µg/mL) for 24 h, and viability was evaluated by Live/Dead assay. **A** and **B** Bars represent relative cell viability expressed as percentage of cells treated with DMSO alone and are means ± SD from two independent experiments. ^a,b^Bars without the same letters differ (*P* < 0.05). **C** and **D** Representative images from fluorescence microscopy of cells following Live/Dead assay. Green colour indicates live IPEC-J2 spheroids, whereas orange colour indicates dead IPEC-J2 spheroids (20× magnification). The size of the spheroids is indicated by the scale bar. **D** Results from one representative Live/Dead assay showing the number of live and dead spheroids, the percentage of live spheroids and the calculated spheroids viability for the different treatments. Abbreviations: AO, Acridine orange; PI, Propidium iodide

Prior to studying the effect of 1,25D_3_ and TM on ER stress and expression of TJP, inflammatory and apoptosis-related genes, the effect of test compounds on the viability of IPEC-J2 cells in 2D and 3D cell cultures was evaluated. In the 2D cell culture, treatment of IPEC-J2 cells with 0.01 to 0.1 µg/mL of TM for 24 h did not impair cell viability when compared to treatment with vehicle alone. At a TM concentration of 0.5 and 1 µg/mL, IPEC-J2 cell viability was decreased by 30%–35% compared to cells treated with vehicle alone (*P* < 0.05, Fig. 2A). Treatment of IPEC-J2 cells with 10 to 500 nmol/L of 1,25D_3_ for 24 h did not reduce IPEC-J2 cell viability when compared to treatment with vehicle alone (Fig. 2B).

In the 3D culture, treatment of IPEC-J2 spheroids with either 0.01 to 0.5 µg/mL of TM or 50 to 500 nmol/L of 1,25D_3_ for 24 h caused no impairment of spheroid viability compared to treatment with vehicle alone (data not shown). At TM concentrations of 1, 2.5 and 5 µg/mL the viability of IPEC-J2 spheroids was reduced by 4%, 15% and 20%, respectively, compared to spheroids treated with vehicle alone (*P* < 0.05, Fig. 2C and D). These findings indicated that IPEC-J2 spheroids are more robust and tolerate higher TM concentrations than IPEC-J2 cells. Based on these results, TM was used at 0.1 µg/mL in the 2D cell culture and at 1 µg/mL in the 3D cell culture, while 1,25D_3_ was used at 50 and/or 100 nmol/L in both the 2D and the 3D cell culture.

### 1,25D_3_ does not mitigate TM-induced ER stress in IPEC-J2 cells in the 2D and the 3D cell culture

**Fig. 3:**
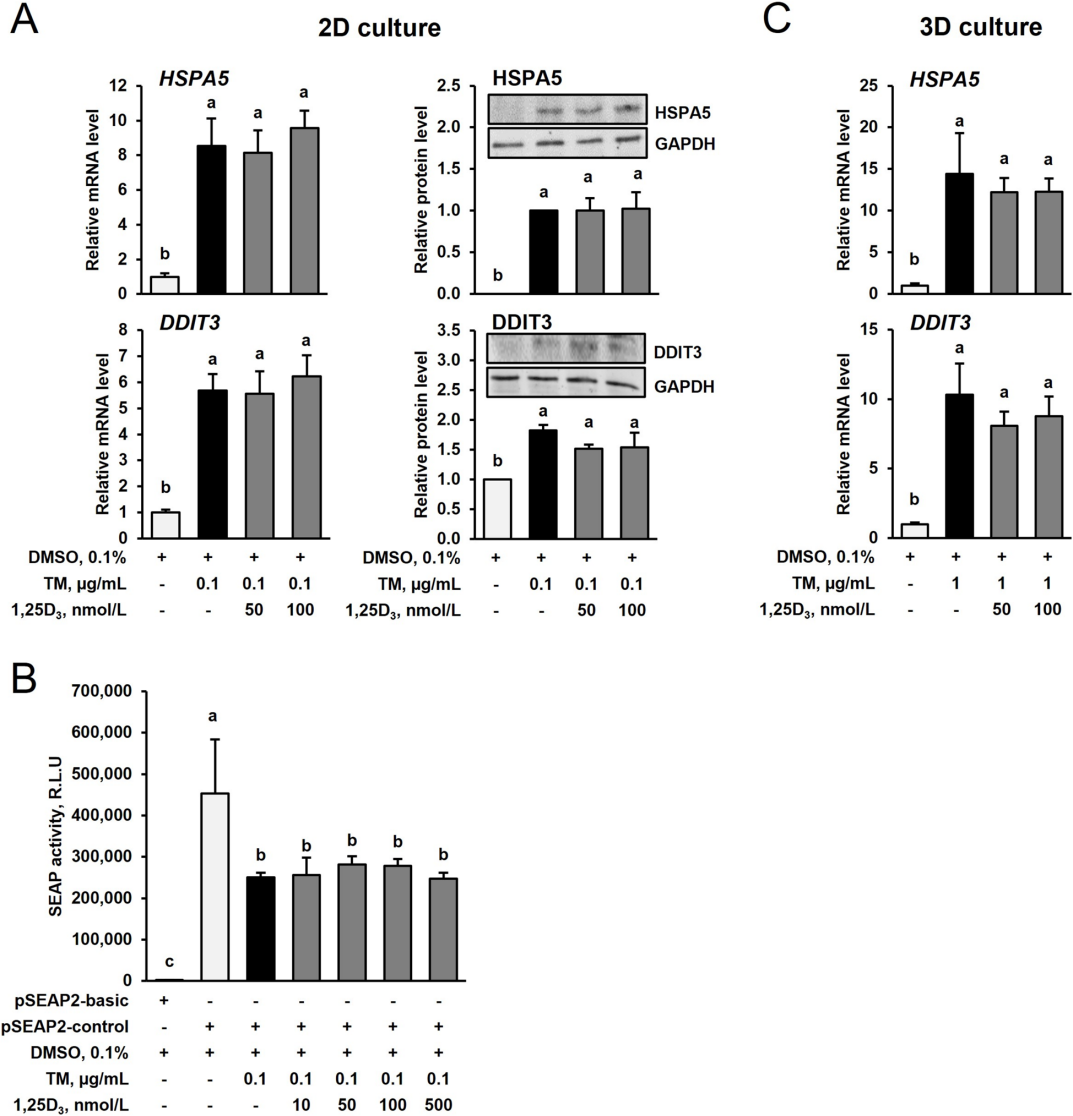
Effect of tunicamycin (TM) and TM and 1,25-dihydroxy-vitamin D_3_ (1,25D_3_) on markers of ER stress in IPEC-J2 cells in the 2D (**A** and **B**) and 3D culture (**C**). For 2D culture, 70%–80% confluent IPEC-J2 cells were treated either without (0.1% DMSO alone) or with TM (0.1 µg/mL) or with TM (0.1 µg/mL) and 1,25D_3_ (50 or 100 nmol/L) for 24 h. For 3D culture, differentiated IPEC-J2 spheroids were treated either without (0.1% DMSO alone) or with TM (1 µg/mL) or with TM (1 µg/mL) and 1,25D_3_ (50 or 100 nmol/L) for 24 h. **A** and **C** Relative mRNA levels and protein levels of HSPA5 and DDIT3 are expressed as fold of control. Representative immunoblots for HSPA5 and DDIT3 including immunoblots for GAPDH as internal control are shown. **B** Secretory alkaline phosphatase (SEAP) activity is shown as relative light units. Data are means ± SD from at least two independent experiments. ^a−c^Bars without the same letters differ (*P* < 0.05)

As expected, treatment of IPEC-J2 cells with TM (0.1 µg/mL) for 24 h markedly increased mRNA (6–8-fold) and protein levels of the ER stress target genes, heat shock protein family A (Hsp70) member 5 (*HSPA5*) and DNA damage inducible transcript 3 (*DDIT3*), relative to treatment with vehicle alone (*P* < 0.05, Fig. 3A). In order to investigate if 1,25D_3_ is able to mitigate ER stress-induction by TM, IPEC-J2 cells were co-treated with TM (0.1 µg/mL) and 1,25D_3_ (50 or 100 nmol/L) for 24 h. Neither the mRNA levels nor the protein levels of HSPA5 and DDIT3 differed between IPEC-J2 cells co-treated with TM and 1,25D_3_ and IPEC-J2 cells treated with TM alone indicating that 1,25D_3_ is not able to mitigate ER stress-induction by TM in the 2D IPEC-J2 cell culture. The above results were confirmed by measurement of SEAP activity, which has been identified as a surrogate marker of ER stress, whereby a decreased SEAP activity is indicative of increased ER stress. In line with this, 24 h-treatment of IPEC-J2 cells transfected with a SEAP expressing plasmid with TM (0.1 µg/mL) showed a 40% reduced SEAP activity indicating increased ER stress as compared to treatment with vehicle alone (*P* < 0.05, Fig. 3B). Co-treatment with TM (0.1 µg/mL) and different concentrations of 1,25D_3_ for 24 h did not modulate SEAP activity as compared to treatment with TM alone indicating that 1,25D_3_ does not mitigate ER stress in 1,25D_3_ in IPEC-J2 cells.

**Fig. 4:**
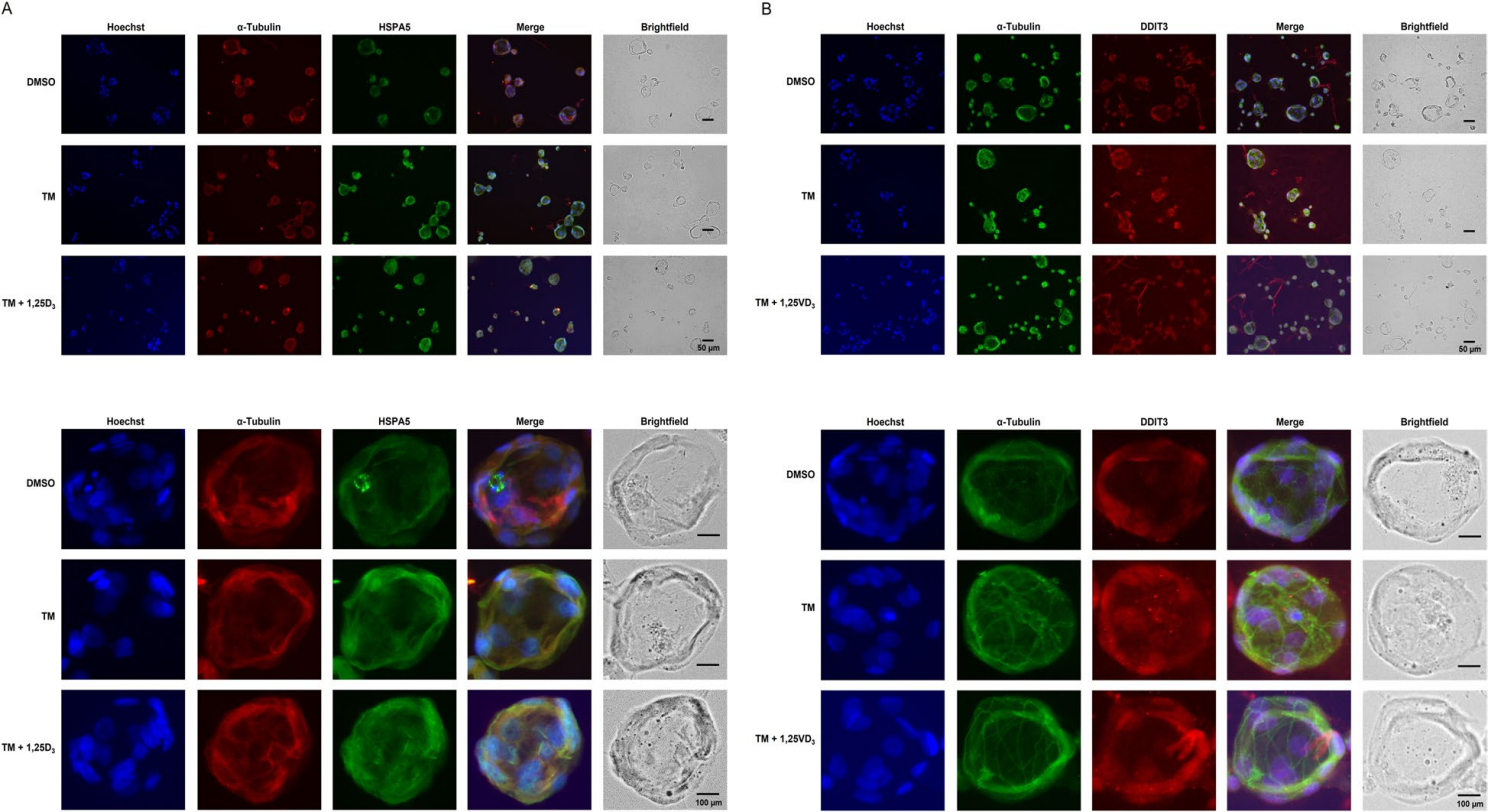
Effect of tunicamycin (TM) and TM and 1,25-dihydroxy-vitamin D_3_ (1,25D_3_) on markers of ER stress in differentiated IPEC-J2 spheroids treated either without (0.1% DMSO alone) or with TM (1 µg/mL) or with TM (1 µg/mL) and 1,25D_3_ (100 nmol/L) for 24 h. Representative images from ICC staining (40× magnification) of differentiated IPEC-J2 spheroids and a single IPEC-J2 spheroid (below) for (**A**) Hoechst (blue), α-Tubulin (red) and heat shock protein family A (Hsp70) member 5 (HSPA5, green), and (**B**) Hoechst (blue), α-Tubulin (green) and DNA damage inducible transcript 3 (DDIT3, red) are shown. A merged image from all three stainings and a brightfield image is also shown. The size of the spheroids is indicated by the scale bar

Similar effects were seen in the 3D IPEC-J2 cell culture. Treatment of IPEC-J2 spheroids with TM (1 µg/mL) for 24 h markedly increased (10–15-fold) *HSPA5* and *DDIT3* mRNA levels (*P* < 0.05, Fig. 3C), but co-treatment of TM with either 50 or 100 nmol/L of 1,25D_3_ for 24 h did not reduce mRNA levels of both ER stress markers (Fig. 3C). ICC-staining of HSPA5 and DDIT3 in IPEC-J2 spheroids largely confirmed the observations from qPCR measurement of *HSPA5* and *DDIT3* mRNA levels that treatment with TM alone (1 µg/mL) for 24 h increased HSPA5 and DDIT3 expression in the IPEC-J2 spheroids, whereas co-treatment with TM and 100 nmol/L of 1,25D_3_ for 24 h did not decrease expression of HSPA5 (Fig. 4A) and DDIT3 (Fig. 4B). These findings clearly showed that the ER stress response of IPEC-J2 cells to TM and 1,25D_3_ is similar in the 2D and 3D cell culture.

### ER stress induces but 1,25D_3_ does not modulate VDR expression in IPEC-J2 cells in the 2D and the 3D cell culture

**Fig. 5:**
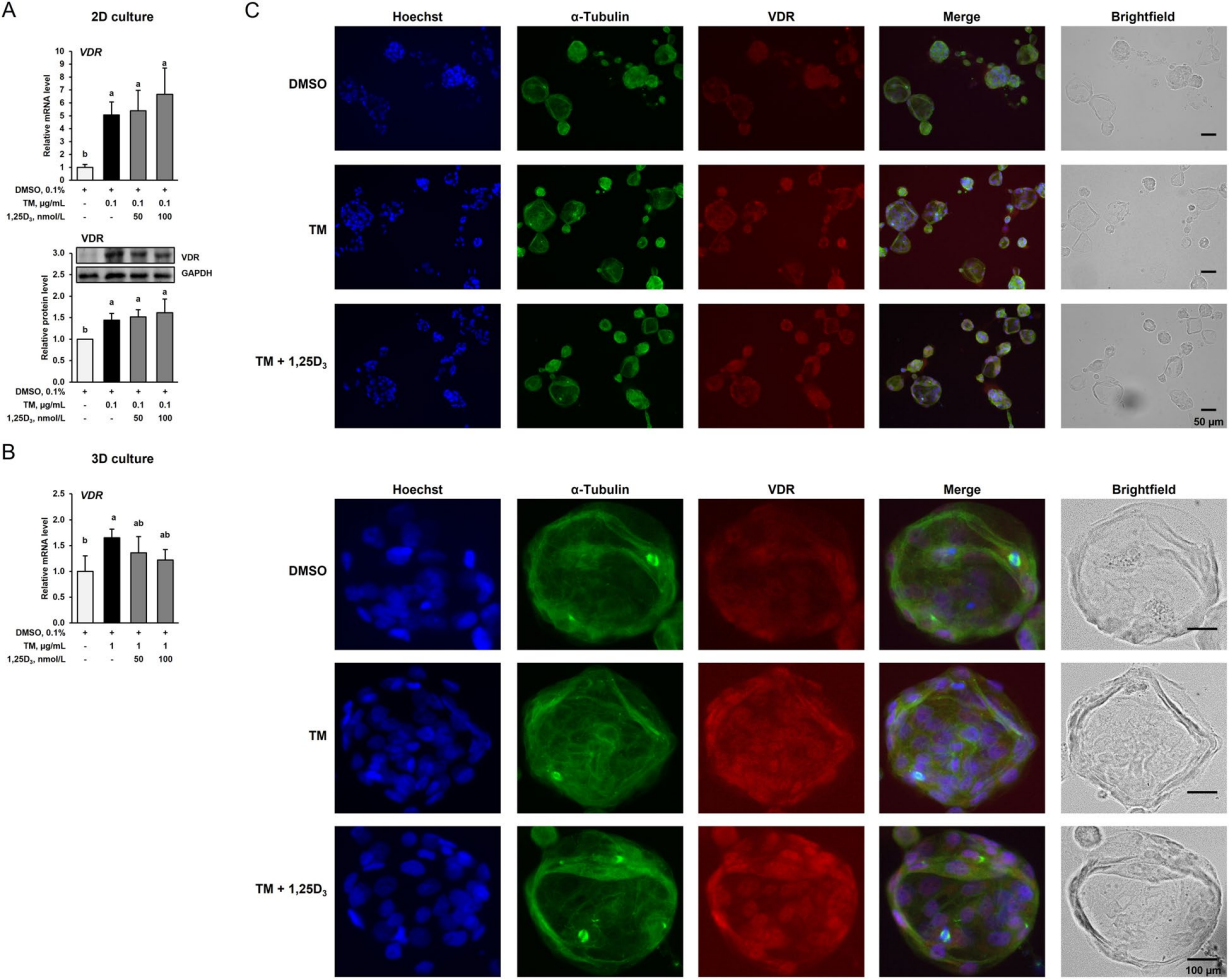
Effect of tunicamycin (TM) and TM and 1,25-dihydroxy-vitamin D_3_ (1,25D_3_) on expression of vitamin D receptor (VDR) in IPEC-J2 cells in the 2D (**A**) and 3D culture (**B**). For 2D culture, 70%–80% confluent IPEC-J2 cells were treated either without (0.1% DMSO alone) or with TM (0.1 µg/mL) or with TM (0.1 µg/mL) and 1,25D_3_ (50 or 100 nmol/L) for 24 h. For 3D culture, differentiated IPEC-J2 spheroids were treated either without (0.1% DMSO alone) or with TM (1 µg/mL) or with TM (1 µg/mL) and 1,25D_3_ (50 or 100 nmol/L) for 24 h. **A** and **B** Relative mRNA levels and protein levels of VDR are expressed as fold of cells treated with DMSO alone. Representative immunoblots for VDR and GAPDH as internal control are shown. Data are means ± SD from at least two independent experiments. ^a,b^Bars without the same letters differ (*P* < 0.05). **C** Representative images from ICC staining (40× magnification) of differentiated IPEC-J2 spheroids and a single IPEC-J2 spheroid (below) for Hoechst (blue), α-Tubulin (green) and VDR (red) are shown. A merged image from all three stainings and a brightfield image is also shown. The size of the spheroids is indicated by the scale bar

The vitamin D receptor (VDR) plays a central role in mediating the effect of 1,25D_3_ in the intestine. In order to investigate if ER stress affects the expression of VDR in IPEC-J2 cells, the *VDR* mRNA level and VDR protein level was determined following 24 h-treatment with TM (0.1 µg/mL). As shown in Fig. 5A, treatment of IPEC-J2 cells with TM caused a marked increase of both *VDR* mRNA (5-fold) and VDR protein (1.5-fold) levels as compared to treatment with vehicle alone (*P* < 0.05). Co-treatment with TM (0.1 µg/mL) and 50 or 100 nmol/L of 1,25D_3_ for 24 h did not affect TM-induced *VDR* mRNA and VDR protein levels in IPEC-J2 cells.

Treatment of IPEC-J2 spheroids with TM (1 µg/mL) for 24 h also increased *VDR* mRNA level (1.6-fold) as compared to treatment with DMSO alone (*P* < 0.05), whereas co-treatment with either 50 or 100 nmol/L of 1,25D_3_ for 24 h numerically but not significantly decreased TM-induced *VDR* mRNA level (Fig. 5B). ICC-staining of VDR in IPEC-J2 spheroids also demonstrated an increased VDR expression following 24 h-treatment with TM alone, whereas co-treatment with TM and 100 nmol/L of 1,25D_3_ for 24 h did not modulate VDR expression (Fig. 5C).

### 1,25D_3_ reduces TM-induced expression of pro-inflammatory and pro-apoptotic genes in IPEC-J2 cells in the 3D cell culture but not in the 2D cell culture

**Fig. 6:**
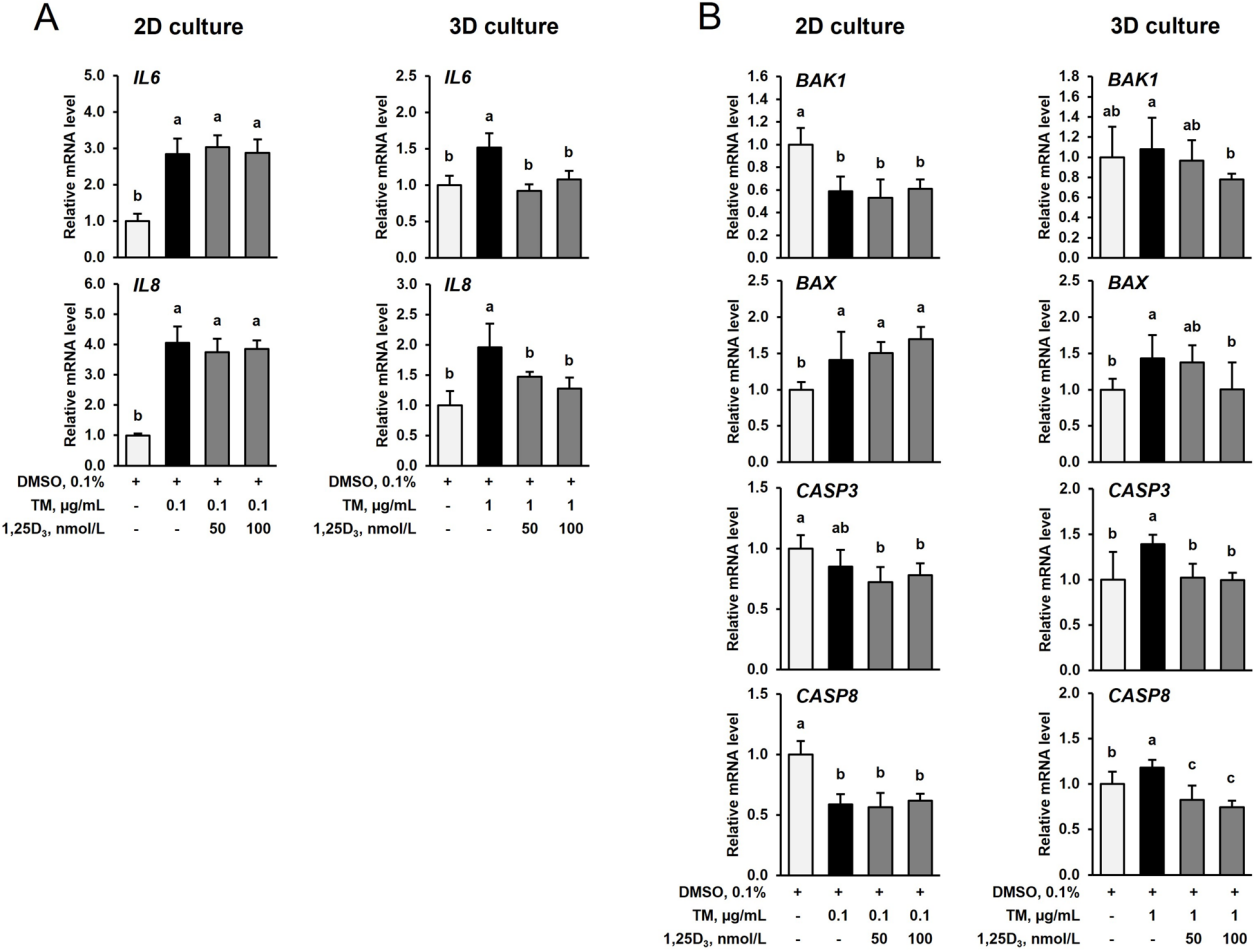
Effect of tunicamycin (TM) and TM and 1,25-dihydroxy-vitamin D_3_ (1,25D_3_) on expression of genes involved in inflammation (**A**) and apoptosis (**B**) in IPEC-J2 cells in the 2D and 3D culture. For 2D culture, 70%–80% confluent IPEC-J2 cells were treated either without (0.1% DMSO alone) or with TM (0.1 µg/mL) or with TM (0.1 µg/mL) and 1,25D_3_ (50 or 100 nmol/L) for 24 h. For 3D culture, differentiated IPEC-J2 spheroids were treated either without (0.1% DMSO alone) or with TM (1 µg/mL) or with TM (1 µg/mL) and 1,25D_3_ (50 or 100 nmol/L) for 24 h. **A** and **B** Relative mRNA levels of interleukin 6 (*IL6*), *IL8*, BCL2 antagonist/killer 1 (*BAK1*), BCL2 associated X, apoptosis regulator (*BAX*), caspase 3 (*CASP3*) and *CASP8* are expressed as fold of cells treated with DMSO alone. Data are means ± SD from at least two independent experiments. ^a,b^Bars without the same letters differ (*P* < 0.05)

In the 2D cell culture, treatment of IPEC-J2 cells with TM (0.1 µg/mL) for 24 h increased mRNA levels of the pro-inflammatory genes interleukin 6 (*IL6*) and *IL8* approximately 3–4-fold compared to treatment with vehicle alone (*P* < 0.05), whereas co-treatment with TM (0.1 µg/mL) and 50 or 100 nmol/L of 1,25D_3_ did not modulate mRNA expression of these genes compared to treatment with TM alone (Fig. 6A). The mRNA levels of the pro-apoptotic genes BCL2 antagonist/killer 1 (*BAK1*), BCL2 associated X, apoptosis regulator (*BAX*), and *CASP8* were differentially affected by 24 h-treatment with TM alone but did not differ between IPEC-J2 cells treated with TM alone and cells co-treated with TM and 50 or 100 nmol/L of 1,25D_3_ in the 2D cell culture (*P* < 0.05, Fig. 6B). In the 3D cell culture, 24 h-treatment of IPEC-J2 spheroids with TM (1 µg/mL) increased the mRNA levels of the pro-inflammatory genes *IL6* and *IL8* (1.5–2-fold, Fig. 6A) and the apoptotic genes *BAX*, *CASP3* and *CASP8* (1.2–1.4-fold, Fig. 6B) compared to treatment with DMSO alone (*P* < 0.05), but co-treatment of the IPEC-J2 spheroids with TM (1 µg/mL) and 50 and/or 100 nmol/L of 1,25D_3_ decreased the mRNA levels of all these genes to a similar level as found in IPEC-J2 spheroids treated with TM alone (Fig. 6A and B). This observation that TM-induced stimulation of inflammation and apoptosis is prevented by 1,25D_3_ in the 3D culture but not in the 2D culture of IPEC-J2 cells indicated that IPEC-J2 cells cultured under 2D conditions are less sensitive to 1,25D_3_ treatment than IPEC-J2 spheroids.

### 1,25D_3_ reduces TM-induced expression of several TJP in IPEC-J2 cells in the 3D cell culture but not in the 2D cell culture

**Fig. 7:**
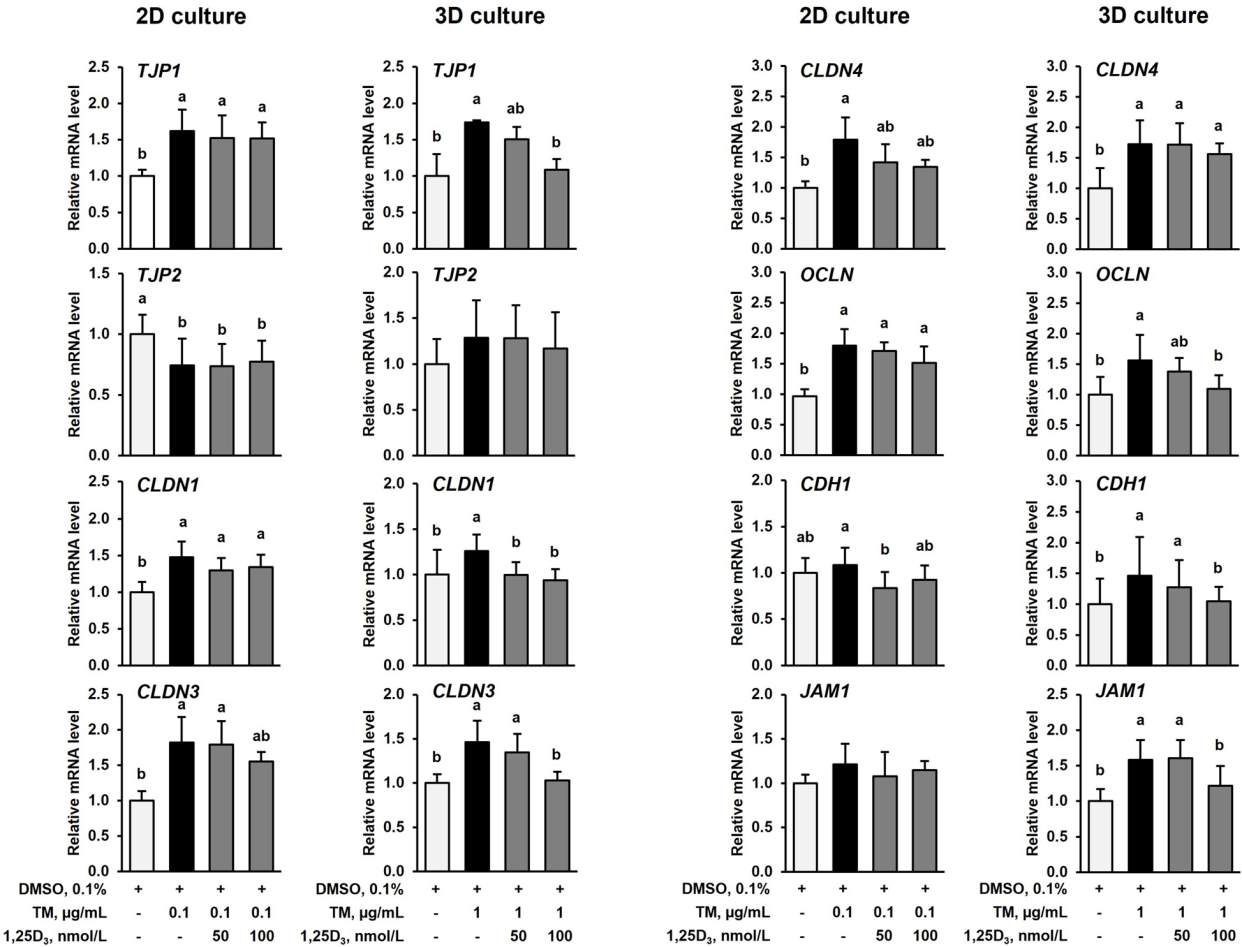
Effect of tunicamycin (TM) and TM and 1,25-dihydroxy-vitamin D_3_ (1,25D_3_) on expression of genes encoding tight junction proteins in IPEC-J2 cells in the 2D and 3D culture. For 2D culture, 70%–80% confluent IPEC-J2 cells were treated either without (0.1% DMSO alone) or with TM (0.1 µg/mL) or with TM (0.1 µg/mL) and 1,25D_3_ (50 or 100 nmol/L) for 24 h. For 3D culture, differentiated IPEC-J2 spheroids were treated either without (0.1% DMSO alone) or with TM (1 µg/mL) or with TM (1 µg/mL) and 1,25D_3_ (50 or 100 nmol/L) for 24 h. Relative mRNA levels of tight junction protein 1 (*TJP1*), *TJP2*, claudin 1 (*CLDN1*), *CLDN3*, *CLDN4*, occludin (*OCLN*), cadherin 1 (*CDH1*) and junctional adhesion molecule 1 (*JAM1*) are expressed as fold of cells treated with DMSO alone. Data are means ± SD from at least two independent experiments. ^a,b^Bars without the same letters differ (*P* < 0.05)

**Fig. 8:**
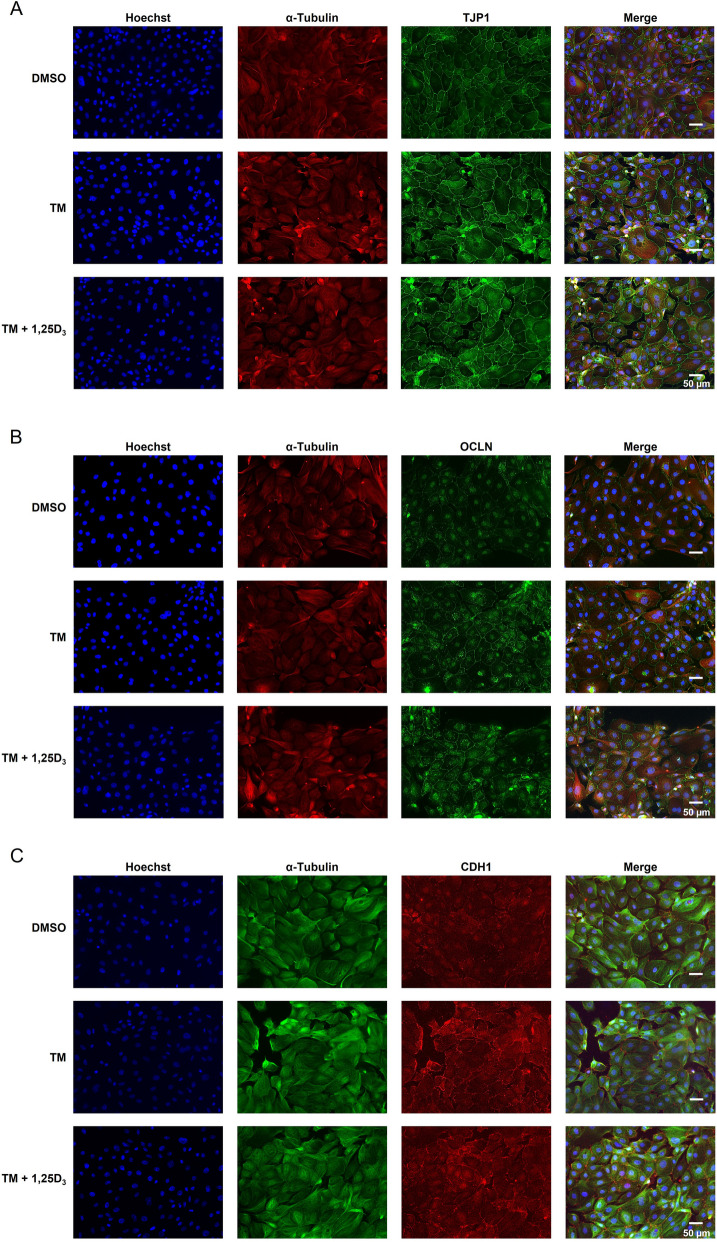
Effect of tunicamycin (TM) and TM and 1,25-dihydroxy-vitamin D_3_ (1,25D_3_) on tight junction protein expression in 2D IPEC-J2 cells treated either without (0.1% DMSO alone) or with TM (0.1 µg/mL) or with TM (0.1 µg/mL) and 1,25D_3_ (100 nmol/L) for 24 h. Representative images from ICC staining (40× magnification) of 2D IPEC-J2 cells for (**A**) Hoechst (blue), α-Tubulin (red) and tight junction protein 1 (TJP1, green), (**B**) Hoechst (blue), α-Tubulin (red) and occludin (OCLN, green), and (**C**) Hoechst (blue), α-Tubulin (green) and cadherin 1 (CDH1, red) are shown. A merged image from all three stainings is also shown. The size of the cells is indicated by the scale bar

Incubation of IPEC-J2 cells under 2D culture conditions with TM (0.1 µg/mL) for 24 h increased mRNA levels of *TJP1*, claudin 1 (*CLDN1*), *CLDN3*, *CLDN4* and occludin (*OCLN*) approximately 1.5–1.8-fold compared to treatment with vehicle alone (*P* < 0.05), whereas co-treatment with TM (0.1 µg/mL) and 50 or 100 nmol/L 1,25D_3_ did not modulate mRNA expression of these genes compared to treatment with TM alone (Fig. 7). The mRNA levels of cadherin 1 (*CDH1*), junctional adhesion molecule 1 (*JAM1*) and *TJP2* were not induced by treatment of IPEC-J2 cells with TM alone compared with vehicle only and did not differ between cells co-treated with TM and 1,25D_3_ and cells treated with TM alone (Fig. 7). ICC-staining of TJP1, OCLN and CDH1 in IPEC-J2 cells demonstrated increased expression of these TJP in response to 24 h-treatment with TM (0.1 µg/mL) alone, whereas expression of TJP1, OCLN and CDH1 did not differ between IPEC-J2 cells co-treated with TM and 100 nmol/L of 1,25D_3_ for 24 h (Fig. 8A–C).

**Fig. 9:**
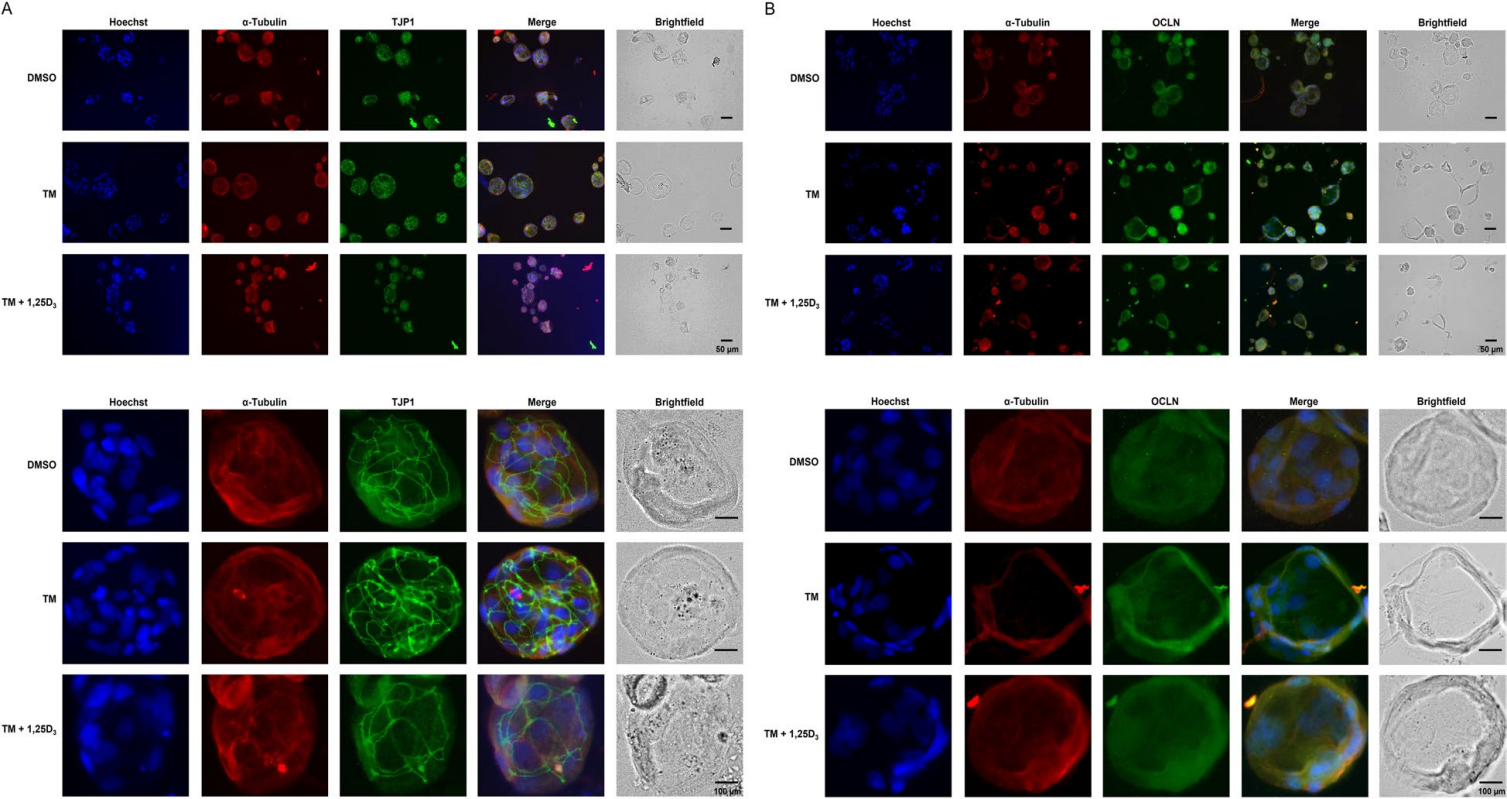
Effect of tunicamycin (TM) and TM and 1,25-dihydroxy-vitamin D_3_ (1,25D_3_) on tight junction protein expression in 3D IPEC-J2 spheroids treated either without (0.1% DMSO alone) or with TM (1 µg/mL) or with TM (1 µg/mL) and 1,25D_3_ (100 nmol/L) for 24 h. Representative images from ICC staining (40× magnification) of differentiated IPEC-J2 spheroids and a single IPEC-J2 spheroid (below) for (**A**) Hoechst (blue), α-Tubulin (red) and tight junction protein 1 (TJP1, green), and (**B**) Hoechst (blue), α-Tubulin (red) and occludin (OCLN, green) are shown. A merged image from all three stainings and a brightfield image is also shown. The size of the spheroids is indicated by the scale bar

Under 3D culture conditions, treatment of IPEC-J2 spheroids with TM (1 µg/mL) increased mRNA levels of *TJP1*, *CLDN1*, *CLDN3*, *CLDN4*, *OCLN*, *CDH1* and *JAM1* approximately 1.3–1.7-fold compared with DMSO alone (*P* < 0.05), but co-treatment with TM and 1,25D_3_ (100 nmol/L) decreased mRNA levels of *TJP1*, *CLDN1*, *CLDN3*, *OCLN*, *CDH1* and *JAM1* to a similar level as found in IPEC-J2 spheroids treated with DMSO alone (Fig. 7). In agreement with these results, ICC-staining revealed increased expression of TJP1 (Fig. 9A) and OCLN (Fig. 9B) in IPEC-J2 spheroids treated with TM alone (1 µg/mL) but decreased expression of TJP1 and OCLN in IPEC-J2 spheroids co-treated with TM and 1,25D_3_ (100 nmol/L) compared to those treated with TM alone.

## Discussion

In the present study, a protocol for developing a 3D culture model of human Caco-2 cells was adopted to porcine IPEC-J2 cells in order to establish a more convincing and meaningful in vitro-model for animal nutrition research. Following this protocol, 5 d of cultivation of IPEC-J2 cells in Matrigel resulted in fully differentiated 3D IPEC-J2 spheroids showing the characteristic spherical architecture with a single layer of IPEC-J2 cells surrounding a central lumen. In addition, proteins that are typically found at the luminal side of the cytoplasm, such as filamentous actin, predominantly localized to the cell surfaces lining the lumens of the IPEC-J2 spheroids indicating polarization of the IPEC-J2 cells. Although the presence of microvilli and the expression of apically located proteins was not investigated, expression of intestinal stem cell marker LGR5, goblet cell marker MUC2, and enteroendocrine cell marker CHGA could not be detected by qPCR and ICC, respectively, (data not shown), suggesting that the differentiated IPEC-J2 spheroids exclusively consisted of enterocytes. When comparing the viability of the differentiated 3D IPEC-J2 spheroids and 2D IPEC-J2 cells in response to the ER stress inducer TM, we found that viability of 2D IPEC-J2 cells was impaired at a lower TM concentration in the culture medium than 3D IPEC-J2 spheroids indicating that IPEC-J2 cells tolerate higher TM concentrations when cultivated in a more tissue-like environment with more pronounced cell–cell and cell–ECM interactions. Due to this, a higher concentration of TM was used in the subsequent experiments with IPEC-J2 spheroids than in those with IPEC-J2 cells.

Induction of ER stress by TM caused a similar response in both IPEC-J2 cells and spheroids, as indicated by strongly increased mRNA levels of the ER stress marker genes *HSPA5* and *DDIT3* but the extent of induction was higher in the IPEC-J2 spheroids than in the IPEC-J2 cells. Induction of ER stress in response to TM was also evident from increased protein levels of HSPA5 and DDIT3 in IPEC-J2 cells and increased ICC staining of HSPA5 and DDIT3 in the differentiated IPEC-J2 spheroids. Since several studies demonstrated that 1,25D_3_ inhibits ER stress in different cell types, such as monocytes, endothelial cells and mammary epithelial cells [8–10], we investigated the hypothesis that 1,25D_3_ (50 and/or 100 nmol/L) inhibits TM-induced ER stress in the IPEC-J2 cell culture. In contrast to the abovementioned studies, our results showed that 1,25D_3_ does not mitigate the effect of TM on the expression of ER stress marker genes in IPEC-J2 cells and IPEC-J2 spheroids indicating that 1,25D_3_ is inefficacious in this cell model regardless of whether cells or spheroids are used. However, it must be mentioned that the inability of 1,25D_3_ to mitigate TM-induced expression of HSPA5 and DDIT3 in the IPEC-J2 culture cannot be explained by a lacking VDR, which is known to mediate many of the 1,25D_3_ effects [19]. Rather, the expression of VDR was even increased by treatment with the ER stress inducer TM in both IPEC-J2 cells and differentiated spheroids while co-treatment with 1,25D_3_ did not modulate VDR expression. This was clearly shown by qPCR, immunoblotting and ICC staining of VDR in the IPEC-J2 cells and spheroids, respectively. This effect of 1,25D_3_ in the IPEC-J2 cell model is in contrast to that in mammary epithelial cells, in which the expression of VDR decreased upon induction of ER stress, whereas 1,25D_3_ increased VDR expression during ER stress. This finding indicates that 1,25D_3_ counter-regulates the inhibitory effect of ER stress on VDR expression in mammary epithelial cells [8]. In addition, in the latter study ER stress altered the expression of hydroxylases involved in regulating 1,25D_3_ levels, such as CYP2R1, CYP27B1 and CYP24A1, in a way favouring an increase of 1,25D_3_ levels, whereas 1,25D_3_ during ER stress modulated the expression of these hydroxylases in a way promoting a decrease of 1,25D_3_ levels. These findings from Wen et al. [8] suggested that 1,25D_3_ acts protective against ER stress in mammary epithelial cells through a mechanism involving an improved responsiveness to 1,25D_3_ through induction of VDR expression. In the present study, the effect of TM and 1,25D_3_ on the expression of CYP2R1, CYP27B1 and CYP24A1 in the IPEC-J2 cell culture was not studied but it is possible that the effects on expression of these hydroxylases differs between mammary epithelial cells and IPEC-J2 cells and spheroids.

An important cellular effect of induction of ER stress is the stimulation of inflammatory signaling pathways including NF-κB signaling [6, 7]. Upon activation of NF-κB, a large set of genes encoding pro-inflammatory products, such as cytokines or chemokines, are induced and contribute to a pronounced burst of inflammatory mediator secretion. In line with this, TM was found to induce the expression of the pro-inflammatory genes *IL6* and *IL8* in both IPEC-J2 cells and IPEC-J2 spheroids. Interestingly, treatment with 1,25D_3_ decreased TM-induced expression of *IL6* and *IL8* in differentiated IPEC-J2 spheroids but not in IPEC-J2 cells demonstrating that the well-documented anti-inflammatory effect of 1,25D_3_ occurs only in the 3D IPEC-J2 cell culture model. Our observation that TM-induced expression of ER stress marker genes was not reduced by 1,25D_3_ in both IPEC-J2 spheroids and IPEC-J2 cells, however, suggests that the anti-inflammatory effect of 1,25D_3_ in the IPEC-J2 spheroids does not involve an inhibition of ER stress. According to recent studies, the anti-inflammatory effect of 1,25D_3_ involves the direct inhibition of key inflammatory pathways. In this regard, 1,25D_3_ was reported to decrease the DNA binding of NF-κB through binding to the VDR [20], which itself can physically interact with IKKβ, thereby, inhibiting NF-κB activation [21]. In addition, evidence has been provided that 1,25D_3_ inhibits the JAK-STAT signaling pathway [22], which also plays important roles in the regulation of immune responses and inflammatory gene expression [23].

Apart from stimulation of inflammatory signaling pathways, ER stress has been shown to activate apoptotic pathways in the case that ER stress is overwhelming and ER homeostasis cannot be re-established. In order to study the effect of TM and 1,25D_3_ on apoptotic pathways, we determined the mRNA levels of apoptosis-related genes, such as the pro-apoptotic *BAX* (former BCL2-associated X protein), the anti-apoptotic *BAK1* (also known as BCL2 antagonist), and the key executors of apoptosis *CASP3* and *CASP8*. As expected, TM increased the mRNA level of *BAX* and the ratio between *BAX* and *BAK1* in both IPEC-J2 cells and IPEC-J2 spheroids, being indicative of induction of apoptosis. In addition, TM induced the pro-apoptotic genes *CASP3* and *CASP8* in IPEC-J2 spheroids but not in IPEC-J2 cells, in which *CASP8* was down-regulated by TM. Apart from the effect of TM on apoptosis-related gene expression, the effect of 1,25D_3_ differed between IPEC-J2 cells and IPEC-J2 spheroids in this regard. While 1,25D_3_ did not modulate TM-induced changes in the expression levels of *BAK1*, *BAX* and *CASP8* in IPEC-J2 cells, the high (100 nmol/L) and, partially, the low concentration (50 nmol/L) of 1,25D_3_ abrogated the stimulatory effect of TM on the expression of *BAX*, *CASP3* and *CASP8* in the IPEC-J2 spheroids. This clearly indicated that 1,25D_3_ is effective in inhibiting TM-induced apoptosis in IPEC-J2 spheroids but not in IPEC-J2 cells. The reason underlying this difference is unclear but it might be possible that the anti-inflammatory activity of 1,25D_3_ in the IPEC-J2 spheroids has enabled the intestinal spheroids to better cope with ER stress-induced stimulation of apoptotic signaling, thereby, enabling an anti-apoptotic effect of 1,25D_3_. In contrast, the lack of an anti-inflammatory activity of 1,25D_3_ in IPEC-J2 cells has probably overcharged the cellular protection mechanisms, thereby, disabling the cells to prevent activation of pro-apoptotic pathways by 1,25D_3_.

The intestinal epithelium plays a vital role in forming a physical and interactive barrier between the intestinal mucosa and the luminal environment. The apical intercellular TJP are multi-protein complexes of the plasma membrane being responsible for the paracellular barrier function, thereby, preventing the transepithelial passage of toxins and microbial components from the intestinal lumen into the portal circulation. Increased intestinal permeability caused by defects in the intestinal barrier is a common feature of several intestinal diseases, such as inflammatory bowel disease, necrotizing enterocolitis and ischemia-reperfusion injury, but is also considered to contribute to various metabolic diseases including fatty liver and metabolic syndrome. The present study demonstrates that TM-induced ER stress is accompanied by an increased expression of several genes encoding TJP, such as *TJP1/ZO1*, *CLDN1*, *CLDN3*, *CLDN4*, *OCLN*, *CDH1* and *JAM1* in both IPEC-J2 cells and IPEC-J2 spheroids, indicating a dysregulation of TJP expression under conditions of ER stress. The stimulatory effect of TM-induced ER stress on the expression of TJP encoding genes including *OCLN*, *CLDN1* and *TJP1* has been also observed in several other studies using epithelial cells [24–26], and has been shown to protect epithelial cells from ischemic injury through preserving epithelial cell architecture, intercellular junctions and cell-substratum interactions [24]. Likewise, a beneficial effect of stem cells on TJP expression and intestinal epithelial permeability in an intestinal organoid model and in a mouse model of necrotizing enterocolitis was found to involve the activation of the ER stress response [27]. This was evident from the observation that inhibition of ER stress using an inhibitor of HSPA5 abolished the effect of stem cells on epithelial TJP expression and barrier function suggesting a regulatory role of the ER stress response for TJP and intestinal barrier function. Our study further revealed that treatment with 1,25D_3_ abrogated the effect of TM on TJP expression in the IPEC-J2 spheroids but not in the IPEC-J2 cells, again showing that the effect of 1,25D_3_ differs between the 3D and the 2D cell culture model. The effect of 1,25D_3_ on TM-induced expression of TJP in the IPEC-J2 spheroids is in line with findings in active ulcerative colitis patients, in which increased protein levels of CLDN1 and CLDN2 and elevated pro-inflammatory cytokine levels (IL6 and IL13) in the colonic mucosa were decreased by 1,25D_3_ [28]. This suggests that 1,25D_3_ exerts an anti-inflammatory effect in the intestine of ulcerative colitis patients which is accompanied by a restoration of normal TJP expression.

Limitations of the study: Despite that the present IPEC-J2 cell culture model better mimics the in vivo-architecture of the small intestinal epithelium than the conventional 2D culture model, a limitation of this model is that the intestinal lumen is located in the interior of the spheroids meaning that treatment compounds, such as 1,25D_3_, are applied from the basolateral side and not from the apical side as the case in the 2D IPEC-J2 cell culture. This may at least partially explain the different outcomes in the effects of 1,25D_3_ between the 2D and the 3D culture conditions. However, in the specific case of 1,25D_3_—the active vitamin D hormone reaching the intestinal epithelium from the blood—the basolateral exposure better reflects the in vivo-situation. A further limitation of the 3D IPEC-J2 cell culture model with regard to the in vivo-transferability is that cell line-derived enteroids, such as IPEC-J2 spheroids, do not fully reflect the cellular complexity of the intestinal epithelium. Although enterocytes are the dominating cell types in the intestinal epithelium, it also contains several other epithelial cell types like the mucin-producing goblet cells and the secretory Paneth cells.

## Conclusion

While the ER stress inducer TM caused a pronounced induction of the ER stress marker genes *HSPA5* and *DDIT3* in both IPEC-J2 cell culture models (6–8-fold and 10–15-fold in the 2D and 3D culture, respectively), the present study clearly shows that the effect of 1,25D_3_ in the IPEC-J2 cell culture is dependent on the culture model applied. Namely, 1,25D_3_ did not inhibit TM-induced expression of genes involved in inflammation (*IL6*, *IL8*), apoptosis (*BAX*) and TJP (*TJP1*, *CLDN1*, *CLDN3*, *OCLN*, *CDH1*, *JAM1*) in the conventional 2D IPEC-J2 cell culture, whereas TM-induced expression of these genes was abrogated by 1,25D_3_ in the more meaningful 3D IPEC-J2 cell culture model. The findings in the more meaningful 3D IPEC-J2 cell model indicate that 1,25D_3_ partially protects from TM-induced activation of pro-inflammatory and pro-apoptotic signaling. Considering the inability of IEC line-derived enteroids to represent the cellular heterogeneity of the intestinal epithelium, future studies have to clarify if the effects of 1,25D_3_ observed in the 3D IPEC-J2 spheroids also occur in other 3D intestinal epithelial structure models, such as enteroids derived from primary intestinal epithelial stem cells [29] or isolated intestinal crypts [30]. The major advantage of the latter enteroids is the presence of different epithelial cell lineages making it a better representative model for the in vivo-situation.

## Supplementary Information

**Additional file 1:** **Table S1** Characteristics of gene-specific primers used for qPCR analysis.

## Data Availability

The datasets used and/or analysed during the current study are available from the corresponding author on reasonable request.

## Abbreviations

1,25D_3_: 1,25-Dihydroxy-vitamin D_3_
2D: Two-dimensional
3D: Three-dimensional
BAK1: BCL2 antagonist/killer 1
BAX: BCL2 associated X, apoptosis regulator
CASP: Caspase
CDH1: Cadherin 1
CLDN: Claudin
DDIT3: DNA damage inducible transcript 3
ECM: Extracellular matrix
ER: Endoplasmic reticulum
HSPA5: Heat shock protein family A (Hsp70) member 5
ICC: Immunocytochemistry
IEC: Intestinal epithelial cells
IL: Interleukin
JAM1: Junctional adhesion molecule 1
MAMP: Microbial-associated molecular patterns
OCLN: Occludin
SEAP: Secreted alkaline phosphatase
TJP: Tight junction proteins
TM: Tunicamycin
UPR: Unfolded protein response
VDR: Vitamin D receptor
