# Supplementary material for: Comparative evaluation of the modulatory role of 1,25-dihydroxy-vitamin D3 on endoplasmic reticulum stress-induced effects in 2D and 3D cultures of the intestinal porcine epithelial cell line IPEC-J2
Source: J Anim Sci Biotechnol. 2024 Nov 10;15:153. doi: 10.1186/s40104-024-01112-6 (PMC11550553; doi:10.1186/s40104-024-01112-6)
Supplement: Supplementary file 1 — Additional file 1: Table S1 Characteristics of gene-specific primers used for qPCR analysis. [file 40104_2024_1112_MOESM1_ESM.docx]

**Additional file 1: Table S1:** Characteristics of gene-specific primers used for qPCR analysis

| Gene | Sequenz (5´-> 3´) | PCR product | NCBI gene |
| --- | --- | --- | --- |
| symbol | forward/reverse | size (bp) | accession nr. |
| *RPS9* | GTC GCA AGA CTT ATG TGA CC | 325 | XM_021094878.1 |
|  | AGC TTA AAG ACC TGG GTC TG |  |  |
| *HSPA5* | TGGAATGACCCGTCTGTGC | 120 | XM_001927795.5 |
|  | TGGTGCAAATGTCTTTGTTTGC |  |  |
| *DDIT3* | CTGAGTCATTGCCTTTCTCCTTCG | 311 | NM_001144845.1 |
|  | ACTTTGTTTCCGTTTCCTGGGTC |  |  |
| *IL6* | AGCAAGGAGGTACTGGCAGA | 257 | NM_001252429.1 |
|  | GTGGTGGCTTTGTCTGGATT |  |  |
| *IL8* | CTGAGAGTGATTGAGAGTGG | 204 | NM_213867.1 |
|  | AGAAATTCTTGGGAGCCACG |  |  |
| *BAK1* | CCC TGT ACG TCT ACC AGA GG | 197 | XM_021098603 |
|  | AAC CAC AGC CAG AAC TAG CA |  |  |
| *BAX* | CTA CTT TGC CAG TAA ACT GGT | 239 | XM_003127290 |
|  | CTC AGC CCA TCT TCT TCC AG |  |  |
| *CASP3* | CTG CCG AGG CAC AGA ATT G | 135 | NM_214131.1 |
|  | CGCCAGGAATAGTAACCAGGTG |  |  |
| *CASP8* | AGA AAG ATG TCC CAG GGG TGA AGA | 121 | NM_001031779.2 |
|  | CAG GGT GAA AGT AGG TTG TGG CA |  |  |
| *TJP1* | GAG GAT GGT CAC ACC GTG GT | 169 | XM_021098827 |
|  | GGA GGA TGC TGT TGT CTC GG |  |  |
| *TJP2* | GCA GAG ACA ACC CCC ACT TT | 117 | XM_001206404 |
|  | CGT TAA CCA TGA CCA CCC GA |  |  |
| *OCLN* | ATG CTT TCT CAG CCA GCG TA | 176 | NM_001163647 |
|  | AAG GTT CCA TAG CCT CGG TC |  |  |
| *CDH1* | CAC GAC TGC AAC AGC CAT GA | 221 | NM_001163060 |
|  | CTG GGT CTG TGA TGA CAA CA |  |  |
| *JAM1* | CCT ATG AGA ACC GAG TTA CC | 164 | NM_001128444.1 |
|  | GAT TGT AGG CTT CGA TGG AG |  |  |
| *CLDN1* | AGA TTT ACT CCT ACG CTG GT | 249 | NM_001244539 |
|  | GCA CCT CAT CAT CTT CCA T |  |  |
| *CLDN3* | CCT ACC ACC GCA AGG ACT AC | 111 | NM_001160075.1 |
|  | GAC TGG TCT CGG ATC CAA GG |  |  |
| *CLDN4* | CGT ACCGAC AAG CCC TAC TC | 119 | NM_0011616337 |
|  | GCA GTC CAG GGA GAA ACC AA |  |  |
| *VDR* | CCT GTC CCT TCA ATG GAG AC | 185 | NM_001097414.1 |
|  | CAG ACT GTC CTT CAA GGC CT |  |  |
